# IBMPFD Disease-Causing Mutant VCP/p97 Proteins Are Targets of Autophagic-Lysosomal Degradation

**DOI:** 10.1371/journal.pone.0164864

**Published:** 2016-10-21

**Authors:** Oznur Bayraktar, Ozlem Oral, Nur Mehpare Kocaturk, Yunus Akkoc, Karin Eberhart, Ali Kosar, Devrim Gozuacik

**Affiliations:** 1 Sabanci University, Faculty of Engineering and Natural Sciences, Molecular Biology, Genetics and Bioengineering Program, Istanbul, 34956, Turkey; 2 Sabanci University, Nanotechnology Research and Application Center, Istanbul, 34956, Turkey; 3 Sabanci University, Faculty of Engineering and Natural Sciences, Mechatronics Engineering Program, Istanbul, 34956, Turkey; 4 Sabanci University, Center of Excellence for Functional Surfaces and Interfaces for Nano Diagnostics (EFSUN), Istanbul, 34956, Turkey; Indian Institute of Science Education and Research, INDIA

## Abstract

The ubiquitin-proteasome system (UPS) degrades soluble proteins and small aggregates, whereas macroautophagy (autophagy herein) eliminates larger protein aggregates, tangles and even whole organelles in a lysosome-dependent manner. VCP/p97 was implicated in both pathways. VCP/p97 mutations cause a rare multisystem disease called IBMPFD (Inclusion Body Myopathy with Paget’s Disease and Frontotemporal Dementia). Here, we studied the role IBMPFD-related mutants of VCP/p97 in autophagy. In contrast with the wild-type VCP/p97 protein or R155C or R191Q mutants, the P137L mutant was aggregate-prone. We showed that, unlike commonly studied R155C or R191Q mutants, the P137L mutant protein stimulated both autophagosome and autolysosome formation. Moreover, P137L mutant protein itself was a substrate of autophagy. Starvation- and mTOR inhibition-induced autophagy led to the degradation of the P137L mutant protein, while preserving the wild-type and functional VCP/p97. Strikingly, similar to the P137L mutant, other IBMPFD-related VCP/p97 mutants, namely R93C and G157R mutants induced autophagosome and autolysosome formation; and G157R mutant formed aggregates that could be cleared by autophagy. Therefore, cellular phenotypes caused by P137L mutant expression were not isolated observations, and some other IBMPFD disease-related VCP/p97 mutations could lead to similar outcomes. Our results indicate that cellular mechanisms leading to IBMPFD disease may be various, and underline the importance of studying different disease-associated mutations in order to better understand human pathologies and tailor mutation-specific treatment strategies.

## Introduction

Inclusion body myopathy with early-onset Paget’s disease and frontotemporal dementia (IBMPFD) is a rare hereditary disorder with multisystem involvement. The disease has an autosomal dominant inheritance, yet it shows adult-onset (Age 20 to 40 years). Myopathy observed in IBMPFD patients may result in disabling muscle weakness, which might eventually be life-threatening due to cardiac and respiratory muscle involvement. Paget’s disease is the cause of bone pain and fractures in the hips, the spine, the skull and in the long bones of arms and legs. In 30% of patients, brain involvement might be manifested as frontotemporal dementia, leading to learning and memory deficits, speech problems, personality changes and defects in social skills. Currently, there is no cure for IBMPFD. The only treatment modality that can be offered to patients is palliative, aiming to alleviate disease symptoms.

The IBMPFD disease locus was mapped to the chromosome 9p21.1-p12 and the disease was associated with mutations of the *VCP/p97* gene. Among disease-causing mutations, arginine 155 (R155) mutation was reported to be the most common one [1–3]. Patients carrying R155C mutations as well as those with R191Q mutations manifested all above-mentioned symptoms of the IBMPFD disease [4–7]. On the other hand, P137L was observed in a Finnish family and the disease affected nine members during 3 generations. In addition to distal leg muscle weakness and atrophy in the anterior compartment muscles, rapidly progressive demetia was observed in this family [8].

VCP/p97 is an essential protein that was conserved from archaea to man (Ter94 in D. melanogaster and CDC48 in S. cerevisiae). The protein is a member of the ATPase associated with various cellular activities (AAA) protein family, and it is one of the most abundant cytosolic proteins. Biological processes involving VCP/p97 include, endoplasmic reticulum-associated degradation (ERAD) pathway, nuclear envelope reconstruction, post mitotic Golgi reassembly and apoptosis [1, 5, 9, 10].

Cytoplasmic aggregates in the affected tissues (brain, bone, muscle) of IBMPFD patients were found to be positive for VCP/p97 and ubiquitin, suggesting that defects in protein degradation pathways might contribute to the pathogenesis and progression of the disease [5, 9–11]. Indeed, mutant VCP/p97 proteins were shown to cause ERAD defects [12–20]. Expression of the IBMPFD disease-associated VCP/p97 mutants R155 and A232 in cells or in mice stimulated autophagic vesicle accumulation [21]. These mutants were reported to cause abnormalities of autophagosome maturation as well, i.e. causing defects of autophagosome-lysosome fusion and autolysosome generation [21, 22]. Moreover, autophagic markers were accumulated in the affected tissues of the VCP/p97 mutant mice [22].

Autophagy is an evolutionarily conserved catabolic process that is responsible for the degradation of cytosolic components, including damaged organelles, protein clumps and aggregates which cannot be degraded by the UPS [23, 24]. Indeed, protein aggregates were observed in various tissues, including brain, bone and muscle of autophagy-defective mice [14, 25]. Proteins and organelles targeted for autophagic destruction are sequestered in double-membrane vesicles called "autophagosomes", that in turn fuse with lysosomes to give rise to "autolysosomes". Lysosomal enzymes, e.g. proteases or lipases, degrade autophagy cargos into their building blocks (e.g. proteins to amino acids) within the acidic environment of autolysosomes. Building blocks are then transported back to cytosol for reuse by the cell, allowing recycling of cellular material.

Around 30 autophagy proteins (ATG proteins) are involved in autophagic vesicle formation and maturation. Autophagic membrane elongation and vesicle completion requires ATG8/MAP1LC3 (shortly LC3) proteins. LC3 processing at the C-terminus by ATG4 creates the free cytosolic LC3-I form of the protein. Upon activation of autophagy pathways, two ubiquitylation-like mechanisms catalyze conjugation of LC3-I to a lipid molecule, a phosphatidylethanolamine (PE), and form the autophagic membrane-associated LC3-II form [26, 27]. Lipidation of LC3 result in a change in its molecular weight from 18 kDa (LC3-I) to16 kDa (LC3-II), and detection of a shift in SDS-PAGE gels is one of the most commonly used tests of autophagy detection. A green fluorescent protein-fused form of LC3 (GFP-LC3) is also widely used to detect autophagy. In this test, recruitment of cytoplasmic diffuse GFP-LC3 protein to autophagosomes results in the formation of GFP-LC3-labeled punctuate structures, allowing quantification of autophagy using fluorescence microscopy [26]. Mitophagy is a special type of autophagy during which old/damaged mitochondria are first ubiquitylated by E3-ligases such as PARK2 (Parkin), and then degraded by the autophagy machinery. Several recent studies point out to the involvement of VCP/p97 in mitophagy [28, 29].

In this study, we identified VCP/p97 P137L mutant proteins as autophagy targets. We confirmed that mere expression of the VCP/p97 P137L mutant was sufficient to induce autophagy under basal fed conditions. In contrast with previous reports using commonly studied mutants (R155 or A232), P137L mutant did not cause an autophagosome maturation defect, and it did not prominently influence autophagic vesicle fusion with lysosomes. Strikingly, autophagy activation by starvation led to a lysosomal activity-dependent preferential degradation of the P137L mutant while sparing the wild-type VCP/p97 protein. Autophagy inducer and mTOR inhibitor Torin-1 showed similar effects. P137L mutant was not an isolated case: We demonstrated that another IBMPFD disease-related mutant, the G157R mutant formed aggregates, induced productive autophagy and it was cleared by autophagic degradation. Our results showed that, in contrast with the current litterature, some VCP/p97mutants are targets of autophagic degradation. Additionally they underline the fact that, even though they all lead to the same disease, different IBMPFD-related VCP/p97 mutations may have different functional outcomes at a molecular and cellular level.

## Materials and Methods

### Plasmids and constructs

VCP/p97 mutants were created using site-directed mutagenesis through mutation of the wild-type VCP/p97 constructs in the pCDNA3-VCP/p97-myc vector as previously described [17]. GFP-LC3 and GFP and RFP tandemly tagged LC3 (tfLC3 or RFP-GFP-LC3) and LAMP1 constructs were also described [7, 26, 30]. pMX-puro-GFP-p62 plasmid (plasmid number: 38277) was reposited from Addgene [31].

### Cell Culture and transfection

Human embryonic kidney (HEK293T) and human osteosarcoma (U2OS) cells were cultured in DMEM (Dulbecco’s modified Eagle’s medium; Biological Industries, Cat. No: BI01-050-1A) supplemented with 10% (v/v) fetal bovine serum (FBS; PAN, Cat. No: P30-3302), antibiotics (penicillin/streptomycin; Biological Industries, Cat. No: 03-031-1B) and 1% (2 mM) L-glutamine (Biological Industries, Cat. No: 03-020-1B). Rat adrenal gland pheochromocytoma (PC-12) cell line was cultured in Ham's F-12K (Kaighn's) Medium (HyClone/Thermo, Cat. No: HY-SH30526.01) supplemented with 17.5% (v/v) fetal bovine serum (FBS; PAN, Cat. No: P30-3302), antibiotics (penicillin/streptomycin; Biological Industries, Cat. No: 03-031-1B) and 1% (2 mM) L-glutamine (Biological Industries, Cat. No: 03-020-1B). All cell lines were cultured under 5% CO_2_ in a 37°C humidified incubator. Transient transfections were performed using standard calcium-phosphate precipitation method. U2OS stable polyclones were generated following selection of transfected cells with 50 μg/ml G418 for 4 weeks.

Starvation-induced autophagy was achieved by incubating cells in EBSS (Earle’s Balanced Salt Solution, Biological Industries, Cat. No: 02-010-1A) for indicated times. Autophagy activation was also activated by incubating cells for 3 hours in a full cell culture medium supplemented with 250 nM Torin-1 (Tocris, Cat. No: 4247). For inhibition of lysosomal activity, cells are treated with 10 μM of E64d (Santa Cruz, Cat. No: Sc201280A) and 10 μM pepstatin A (Sigma, Cat. No: P5318) for 4 hours or 10 μM of cloroquine (Sigma, Cat. No: C6628) for 40 min. 1 μg/ml Tunicamycin (Sigma, Cat. No: T7765) was used as a ER-stress inducer for 1,2,4 and 8 hours. 1μM Cycloheximide (Sigma, Cat. No: C1988) was used to inhibit protein synthesis.

### Primary myoblast cell isolation and culture

Primary myoblast isolation protocol was adapted from Springer et al. [32]. Briefly, C57B6 male mice were sacrificed, limb and pectoral muscles were removed and diced in DMEM. For 1 ml medium, 500 μl Liberase enzyme (Liberase TL Research Grade, Roche Cat. No: 05401020001. 2,5 mg/ml stock) was added and tissues were incubated at 37°C degree for 90 min under rotation. Following 3 washes in DMEM plus 5% FBS, myoblasts were cultured in DMEM supplemented with 10% (v/v) fetal bovine serum (FBS; PAN, Cat. No: P30-3302), antibiotics (penicillin/streptomycin; Biological Industries, Cat. No: 03-031-1B) and 1% (2 mM) L-glutamine (Biological Industries, Cat. No: 03-020-1B).

### Immunoprecipitation and Immunoblotting

For immunoprecipitation, cells were transfected in 10 cm plates (2 x 10^6^ cells/plate) cultured for 48 hours. After transfection, cells were washed 3 times with cold PBS by centrifugation (13200 rpm, 5min, 4°C). The resulting pellet was resuspended in cold RIPA buffer (1M Tris-HCl, pH 7.6, 5% deoxycholic acid, 10% NP-40, and 0.5 M NaCl) supplied with protease inhibitior cocktail (Sigma, Cat. No: P8340) and 1 mM phenylmethylsulfonyl fluoride (PMSF; Sigma-Aldrich, P7626). Cell lysate was cleared by centrifugation at 13200 rpm for 15 min at 4°C and protein concentrations were estimated by using the Bradford Reagent (Sigma Aldrich, Cat. No: B9616). 1mg of protein was loaded onto the Myc-beads (Sigma-Aldrich, Cat. No: A7470). After several washing with cold PBS and RIPA buffer, the samples were incubated for over-night at 4°C on a rotator. In order to remove non-specific binding, the samples were washed 3 times with RIPA and then analyzed by SDS-PAGE. Immunoblotting was performed as previously described [33]. Briefly, 30 μg of protein was loaded into 15% SDS polyacrylamide gels. Following transfer, nitrocellulose membranes (Millipore, Cat. No: IPVH00010) were blocked with 5% nonfat milk in PBST (3.2 mM Na_2_HPO4, 0.5 mM KH_2_PO_4_, 1.3 mMKCl, 135 mMNaCl and 0.05% Tween 20, pH 7.4) for 1 h and then incubated with 3% BSA containing PBST solution with primary antibodies: Anti-myc (Origene, Cat. No: TA100010), anti-LC3B (Novus, 2331), anti-VCP/p97 (BD Transduction Laboratories, Cat. No: 612183), anti-GFP (Roche, Cat. No: 11814460001), anti-ubiquitin (Cell Signaling Technology, Cat. No: P4D1) (Santa Cruz, Cat. No: sc8017) antibodies were used. Anti-β-ACTIN (Sigma-Aldrich, Cat. No: A5441) antibody was used as loading control. Anti-mouse (Cat. No: 115035003) and anti-rabbit (Cat. No: 111035144) secondary antibodies were purchased from Jackson Laboratories. ImageJ software was used for band intensity quantification [34].

### GFP-LC3 and RFP-GFP-LC3 autophagy analyses

Cytoplasmic puncta formation by otherwise soluble GFP-fused LC3 protein is used as a method to quantify autophagy activation under a fluorescence microscope [26]. Briefly, cells were cultured on top of poly-L-Lys coated coverslides. 48 hours after transfection, cells were fixed with ice-cold 4% PFA in PBS for 30 min. GFP-LC3 dot analysis was performed. Coverslides were analyzed under fluorescence microscope (Olympus BX60, Japan) under 60x magnification. Minimum 150 cells per condition were analyzed. Cells containing more than 15 dots were considered as autophagy positive. Percentage of GFP- LC3 positivity was expressed as a percentage of GFP-LC3 dot positive cells within the total transfected cell population.

RFP-GFP-LC3 dot numbers were determined under a fluorescence microscope (Olympus BX60, Japan) using 60x magnification. Dot numbers were analyzed in 50 cells for each experimental point. Autophagosomes gave both RFP and GFP signals, while autolysosomes were defined as RFP positive dots. Number of autolysosomes was calculated by subtracting GFP positive dot numbers from RFP positive dot numbers.

### Immunofluorescence and confocal microscopy analyses

Cells were seeded in 12-well plates (50,000 cells/well) in poly-L-Lysine (Sigma, Cat. No: P8920) coated coverslides. 48 hours after transfection, cells were fixed in 4% PFA for 30 min, washed with PBS and permeabilized using 0.1% Saponin and 0.1% BSA in PBS. After PBS washes, coverslides were incubated with the primary antibody for 1h at RT. Then, coverslides were incubated with Alexa594-(Molecular probes, Cat. No: 927075) or Alexa488-(Molecular probes, Cat. No: 948490) conjugated secondary antibodies for 1h at RT. Coverslides were mounted using 50% glycerol in 1X PBS at RT. Pictures were taken using the Zen software (Carl-Zeiss, Germany). Intracellular localizations of proteins were analyzed under a fluorescent microscope (Olympus BX60, Japan) and/or using confocal microscopy (Carl-Zeiss LSM 710, Germany). Objective lens of confocal microscope is plan-apochromat, 63x/1.40 Oil DIC M27. Objective lens of flourescent microscope is plan-apochromat 60x/1.42 Oil.

### Cell Fractionation

To separate cell lysate into soluble and insoluble fractions, cells were lysed in Triton X-100 lysis buffer on ice for 30 min, then centrifuged at 14,000 rpm for 10 min. The resulting pellet was lysed in the SDS lysis buffer as the insoluble fraction. The supernatant was used as the soluble fraction.

### Statistical analyses

Statistical analyses were performed using Student’s two tailed t-test. Data were represented as means of ±S.D. of 3 independent experiments. Values with p< 0.05 were considered as significant.

## Results

**Fig 1:**
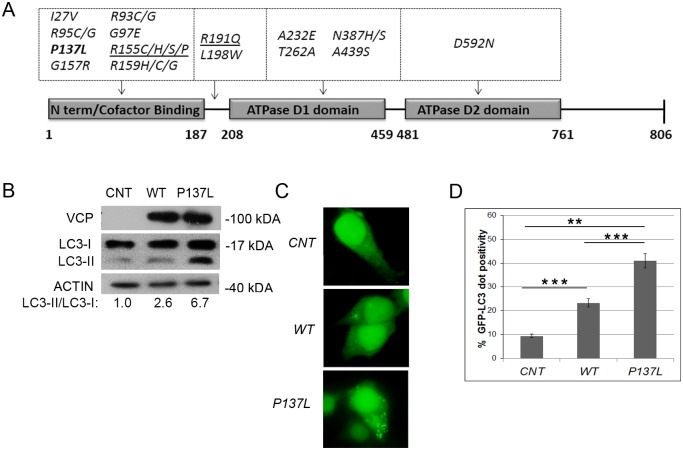
VCP/p97 mutant P137L induced autophagy. (A) Primary structure of VCP/p97 protein and IBMPFD-related mutations. Major protein domains were shown. Bold, P137 mutant; underlined, R155, R191 and A232 mutants. (B) HEK293T cells were transfected with empty vector (CNT), MYC-tagged wild type (WT) or mutant VCP/p97 (P137L) and western blot analysis was performed using MYC, LC3 and ACTIN antibodies. ACTIN was used as loading control. LC3-I and LC3-II band intensity was quantified on scans of immunoblot films using Image J software. LC3-II/LC3-I ratios were determined after subtraction of non-band containing area intensities, and normalized to CNT condition. (C and D) WT or P137L mutant VCP/p97 were co-transfected with GFP-tagged LC3 in HEK293T cells and dot formation was visualized (C) and quantified (D). Data were shown as mean ± SD of independent experiments (n = 3) *** p<0.01.

Various mutants of VCP/p97 were associated with the IBMPFD disease. Majority of reported mutations were shown to affect the N-terminal cofactor-binding domain of the VCP/p97 protein, but other mutations involving ATPase domains and linker regions were observed as well (Fig 1A). We screened the effect of expression of a number of IBMPFD-causing mutants of VCP/p97 on autophagy, and discovered that most mutants stimulated autophagy-associated LC3-II formation. Among them, P137L mutant VCP/p97 was one of the strongest inducers of autophagy in our analyses. In contrast with R155, A232 and R191 mutants of VCP/p97, the P137 mutant was shown to be defective in binding co-factors Npl4, Ufd1 and p47, components necessary for proper functioning of the protein in pathways including ERAD [17].

Indeed, expression of the P137L mutant protein resulted in a prominent LC3 shift and autophagy-associated LC3-II formation compared to wild-type (WT) VCP/p97 (Fig 1B). To further validate these results, GFP-LC3 dot formation was used as another autophagy test. Overexpression of P137L mutant protein led to a strong and significant increase in autophagic GFP-LC3 dot formation in cells expressing P137 mutant VCP/p97 compared to controls (Fig 1C and 1D). All these results showed that the P137L mutant is a potent activator of autophagy.

**Fig 2:**
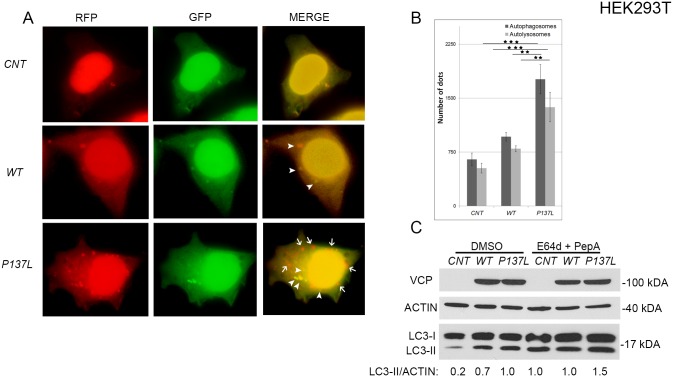
VCP/p97 P137L mutant induced both autophagosome and autolysosome formation in HEK293T cells. (A) Cells expressing empty vector (CNT), wild type (WT) or mutant VCP/p97 (P137L) were co-transfected with RFP-GFP-LC3 fusion construct and dot formation was visualized under fluorescent microscope. Arrow heads indicate autophagosomes and arrows indicate autolysosomes. (B) The graph shows the number of autophagosomes and autolysosomes in WT and P137L mutant expressing cells. Data were shown as mean ± SD of independent experiments (n = 3). **, p<0.05; ***, p<0.01 (C) Cells were transfected with CNT, and MYC-tagged WT or P137L mutant in the absence or presence of lysosomal inhibitors E64d and Pepstatin A (PepA) and western blot analysis was performed. ACTIN was used as loading control. ImageJ software was used for the quantification of band intensities.

**Fig 3:**
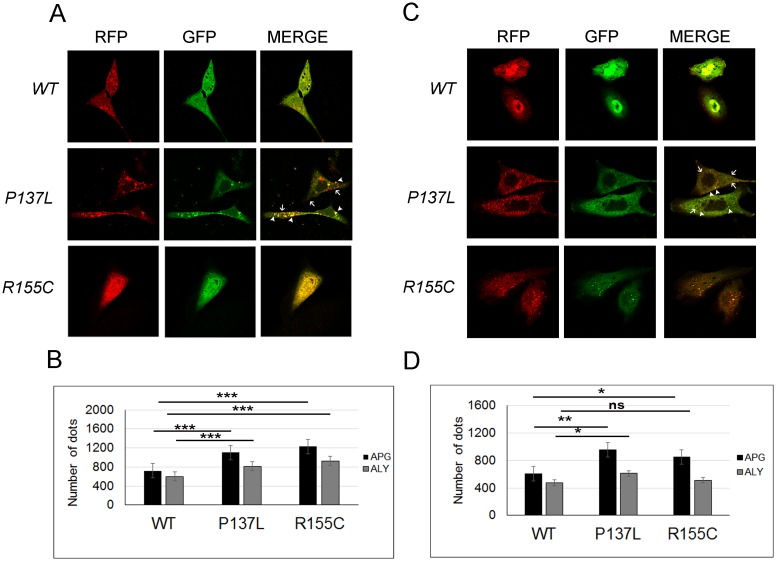
VCP/p97 P137L mutant induced both autophagosome and autolysosome formation in U2OS and PC-12 cells. (A and C) Cells expressing wild type (WT), mutant(P137L or R155C) VCP/p97 were co-transfected with RFP-GFP-LC3 fusion construct and dot formation was visualized in U2OS (A) and PC-12 (C) cells. Arrow heads indicate autophagosomes whereas arrows indicate autolysosomes. (A and C) The graphs show the number of autophagosomes and autolysosomes in U2OS (B) and PC-12 (D) cells. Data were shown as mean ± SD of independent experiments (n = 3). * p<0.05, ** p<0.01, *** p<0.001.

Commonly studied IBMPFD-related mutants of VCP/p97 R155C, R155H and A232E, were shown to interfere with autophagic vesicle maturation and lysosomal fusion, leading to the accumulation of autophagic vesicles and to the perturbation of the autophagic flux [21, 22]. To study a possible effect of the P137L mutant on autophagosome-autolysosome fusion, we used RFP-GFP-LC3 fusion protein as a marker. Here, GFP and RFP both mark autophagosomes. But following fusion with lysosomes, the GFP part is quenched in the acidic environment, while the RFP still emits fluorescence, marking the autolysosomal compartment. Using this method, autophagosome-autolysosome numbers can readily be calculated [7]. RFP-GFP-LC3 analyses showed that both autophagosome (green) and autolysosome (red minus green) numbers were increased upon P137L mutant overexpression in HEK293T cells (Fig 2A). The increase in autophagosome and most strikingly autolysosome numbers were significantly higher in cells overexpressing the P137L mutant, compared to negative control and WT VCP/p97 (Fig 2B). Of note, while both proteins were expressed in comparable amounts, the increase in autolysosome numbers was almost double in P137L mutant expressing cells compared to cells with the WT VCP/p97 (Fig 2B). Moreover, autophagic LC3-II levels were markedly increased in P137L expressing cells upon inhibition of lysosomal proteases using E64d and Pepstatin A. During autophagy, LC3-II itself is delivered to autolysosomes and it is normally degraded in this compartment through the action of lysosomal hydrolases. Therefore, accumulation of LC3-II upon lysosomal protease inhibition indicated that autophagosome maturation and autolysosomal activity were intact (Fig 2C). Similar results were also obtained in U2OS (Fig 3A and 3B) and PC-12 cells (Fig 3C and 3D). Suprisingly, R155C mutant did also induce a significant amount of autolysosome formation in the U2OS cell line. Moreover, following treatment of the same cell types with chloroquine (a lysosomotropic agent that prevents lysosomal acidification and autophagosome-lysosome fusion), mutant proteins led to further accumulation of autophagosomes, indicating that the dominant effect of mutant proteins indeed was stimulation of autophagy rather than blockage of autophagosome-lysosome fusion (S1A–S1D Fig). These results showed that, the P137L mutant did not have a prominent effect on autophagosome maturation and autolysosome formation in HEK293T cells, and more importantly in IBMPFD disease-relevant U2OS bone-derived cells and PC-12 neuronal cells.

While analyzing intracellular localization of VCP/p97 mutants, we realized that, in contrast with the WT VCP/p97 and commonly tested R155C mutant showing a rather diffuse cytoplasmic pattern, the P137L mutant was localized to punctate structures in the cytosol (S2A Fig and see the reference [17]). This punctuate pattern of the P137L mutant was also observed in primary myoblast cells in culture (S2B Fig). Additionally, while the WT VCP/p97 protein was mostly found in the soluble fraction of cells, a major fraction of the P137L mutant protein was insoluble in HEK293T (S2C Fig), U2OS (S2D Fig) and PC-12 (S2E Fig) cells. Moreover, these aggregates were found to colocalize with the autophagy adapter/receptor p62 under basal and starved conditions (S2F Fig).

**Fig 4:**
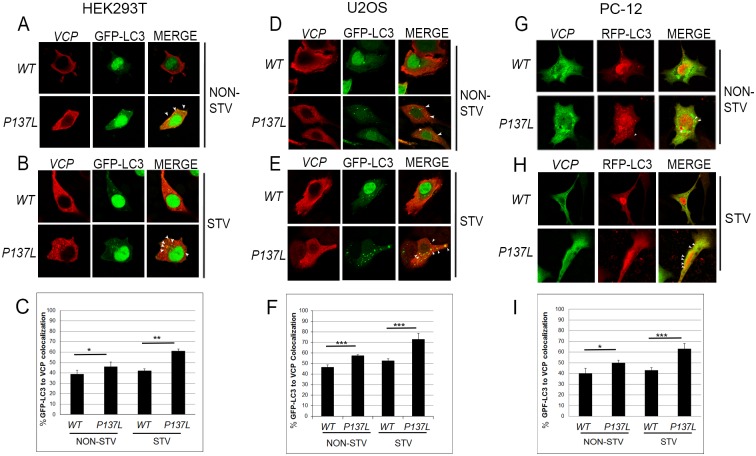
Mutant VCP/p97 colocalized with the autophagic vesicle marker LC3 during starvation. HEK293T (A-C), U2OS (D-F) and PC-12 (G-I) cells expressing wild type (WT) or mutant VCP/p97 (P137L) were co-transfected with GFP-LC3 and colocalization was analyzed by confocal microscopy under basal/non-starved (NON-STV) (A, D, G) or starved (STV) (B, E, H) conditions. Overexpressed MYC-VCP was indirectly immunostained using anti-MYC antibodies. Arrowheads indicate colocalized dots in (A,B),(D,E) and (G,H). Quantification of co-localized dots in HEK293T (C), U2OS (F) and PC-12 (I) cells. Minimum 40 cells were counted under each condition, and quantification was expressed as a percentage of colocalization positive cells within a total transfected cell population. Data were shown as mean ± SD of independent experiments (n = 3). * p<0.05, ** p<0.01.

To determine the intracellular localization of P137L punctuate structures, we performed confocal microscopy analyses. We observed that P137L partially colocalized with the GFP-LC3 autophagic vesicle marker under basal/non-starved conditions in HEK293T (Fig 4A), U2OS (Fig 4D) and PC-12 cells (Fig 4G). Importantly, upon induction of autophagy by starvation, the overlap between LC3 and P137L mutant puncta significantly increased in all tested cell types (Fig 4B, 4C, 4E, 4F, 4H and 4I). In these studies, WT VCP/p97 was used as a control and showed minimal colocalization with the autophagy marker LC3 (Fig 4A–4I).

**Fig 5:**
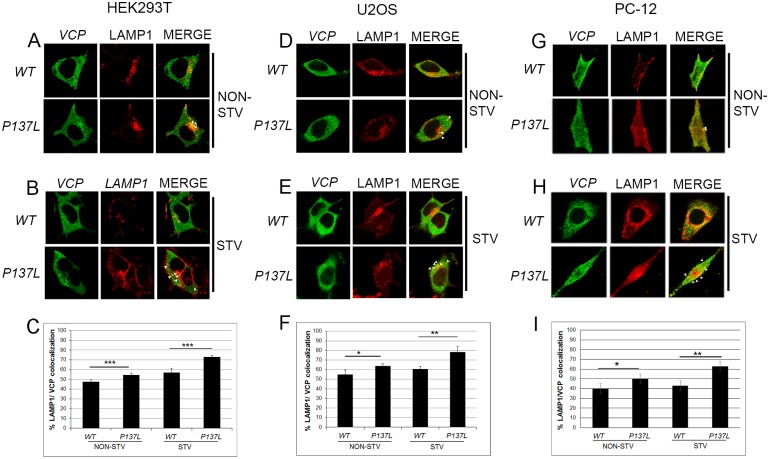
Mutant VCP/p97 colocalized with the lysosome/autolysosome marker LAMP1 during starvation. HEK293T (A-C), U2OS (D-F) and PC-12 (G-I) cells expressing wild type (WT) or mutant VCP/p97 (P137L) were co-transfected with LAMP1-RFP and colocalization was analyzed by confocal microscopy under basal/non-starved (NON-STV) (A, D, G) or starved (STV) (B, E, H) conditions. Overexpressed MYC-VCP was indirectly immunostained using anti-MYC antibodies. Arrowheads indicate colocalized dots in (A,B),(D,E) and (G,H). Quantification of co-localized dots in HEK293T (C), U2OS (F) and PC-12 (I) cells. Minimum 40 cells were analyzed under each condition, and quantification was expressed as a percentage of colocalized positive cells within the total transfected cell population. Data were shown as mean ± SD of independent experiments (n = 3). * p<0.05, ** p<0.01.

LAMP1 is an integral membrane protein of autolysosomes. Confocal analyses using LAMP1 as an autolysosome marker showed that while WT VCP/p97 protein overexpression resulted in a minimal colocalization, P137L mutant VCP/p97 partially colocalized with autolysosomes under non-starved conditions in HEK293T (Fig 5A and 5C), U2OS (Fig 5D and 5F) and PC-12 cells (Fig 5G and 5I). Strikingly, P137L colocalization with LAMP1 was significantly increased following autophagy induction by starvationin the tested cell lines (Fig 5B, 5C, 5E, 5F, 5H and 5I).

**Fig 6:**
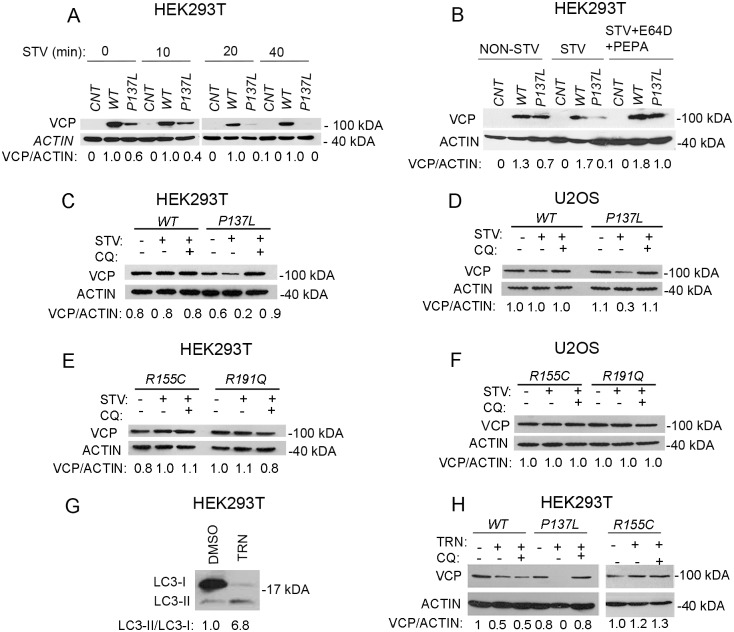
P137L mutant VCP/p97, but not the WT VCP/p97 or R155C and R191Q mutants, was a target of starvation-induced autophagy. (A) HEK293T cells were transfected with empty vector (CNT), MYC-tagged wild type (WT) or mutant VCP/p97 (P137L) and then starved for indicated time points. (B and C) HEK293T cells and (D) U2OS cells expressing empty vector (CNT), wild type (WT) or mutant (P137L) VCP/p97 were starved for 40 min in the absence or presence of lysosomal inhibitors E64d/Pepstatin A (E64D/PEPA) or chloroquine (CQ). (E) HEK293T cells and (F) U2OS cells expressing other VCP/p97 mutants (R155C) and (R191Q) were starved for 40 min in the absence or presence of lysosomal inhibitor chloroquine (CQ). (G) U2OS cells expressing wild type (WT) or mutant VCP/p97 (P137L) and (R155C) were starved for 40 min in the absence or presence of lysosomal inhibitor chloroquine (CQ). (H) U2OS cells were treated with carrier (DMSO) or Torin-1 (TRN) and western blot analysis was performed by using LC3 antibody. (I) Cells were transfected with wild type (WT) and mutant (P137L or R155C) VCP/p97 and treated with TRN in the presence or absence of CQ. ACTIN was used as loading control. Image J software was used for the quantification of band intensities. Western blot analysis was performed by using MYC and ACTIN antibodies. ACTIN was used as loading control. Image J software was used for the quantification of band intensities.

Results obtained so far showed that P137L mutant VCP/p97 could be a target of autophagic degradation. To test this hypothesis, we overexpressed WT VCP/p97 or the P137L mutant in cells, and analyzed the effect of autophagy activation on VCP/p97 protein levels. Upon minutes of starvation, the amount of P137L mutant protein gradually decreased in HEK293T cell extracts, but WT VCP/p97 protein levels remained relatively stable (Fig 6A). To further confirm that autophagic protein degradation was responsible for the observed decrease in P137 protein levels, we performed starvation experiments in the absence or presence of lysosomal inhibitors. Addition of lysosomal protease inhibitors E64d/PepstatinA reversed the effect of starvation on P137L protein levels, and resulted in its accumulation, while WT VCP/p97 levels were relatively stable (Fig 6B). Another lysosomal inhibitor chloroquine, gave similar results confirming that P137L degradation was dependent on lysosomal activity (Fig 6C). A decrease in P137L mutant protein levels upon starvation, and a lysosome-inhibition-dependent accumulation of the mutant protein were also observed in U2OS cells (Fig 6D).

To test whether VCP/p97 mutants R155C and R191Q were degraded by autophagy, mutant proteins were overexpressed in cells, and starvation assays were performed in the presence or absence of the lysosomal inhibitor chloroquine. Different from the P137L mutant, VCP/p97 mutants R155C and R191Q were not degraded upon autophagy activation by starvation, and consequently, inhibition of the autolysosomal activity by chloroquine did not result in their accumulation in either HEK293T (Fig 6E) or U2OS (Fig 6F) cells.

In addition to starvation, various agents and chemicals were shown to induce autophagy. Torin-1 is a specific inhibitor of the mTOR kinase and a classical inducer of autophagy. Therefore, we tested the effect of autophagy-induction by this compound on P137L mutant protein levels. As shown in Fig 6G, Torin-1 was able to stimulate LC3-II formation and activate autophagy under our experimental conditions. In line with starvation assays, following Torin-1 treatment, P137L mutant protein levels were decreased upon autophagy activation, while WT VCP/p97 levels and R155C mutant levels remained constant (Fig 6H). Moreover, inhibition of the lysosomal activity in Torin-1-stimulated cells by chloroquine, blocked P137L mutant protein degradation and led to its accumulation.

To exclude the possibility that P137L mutant VCP/p97 is a short-lived protein that is prone to degradation in cells following expression, we treated cells with cycloheximide, a protein synthesis inhibitor, and compared WT and P137L mutant VCP/p97 protein levels, and observed no obvious difference in stability (S3 Fig). Therefore, P137L mutant protein was a target of autophagy and autolysosomal degradation, while WT VCP/p97 or other relatively soluble mutants of VCP/p97 were not.

VCP/p97 proteins work as homo-hexamers. We wondered whether P137L mutant VCP/p97 could still interact with its WT counterpart and lead to the perturbation of its function. To address this question, we co-expressed wild-type and mutant VCP with different tags (GFP-tagged WT VCP/p97 and MYC-tagged P137L) in cells, and checked whether they interacted. Immunoprecipitation experiments showed that, P137L mutant could still bind to WT VCP/p97 (S4 Fig) and possibly took part in VCP/p97 hexamers to a certain extent. Since the WT VCP was minimally degraded following induction of autophagy (Fig 6), our data indicate that most P137L mutant aggregates did not include WT proteins. In a previous work, we showed that the P137L mutant VCP/p97 was unable to bind its ERAD-related co-factors, and it interfered with the ERAD-mediated degradation of CFTRΔF508 and Tyrosinase C89R mutant proteins [17]. In line with this, expression of the P137L mutant VCP/p97 resulted in the accumulation of ubiquitylated proteins in cells treated with ER stress inducer tunicamycin (S5 Fig). Therefore, non-functional WT/mutant hetero-hexameric VCP complexes, mutant protein insoluble aggregates as well as possible saturation of the UPS by misfolded mutant and endogenous proteins could be in the origin of cellular defects observed in IBMPFD diseased cells.

**Fig 7:**
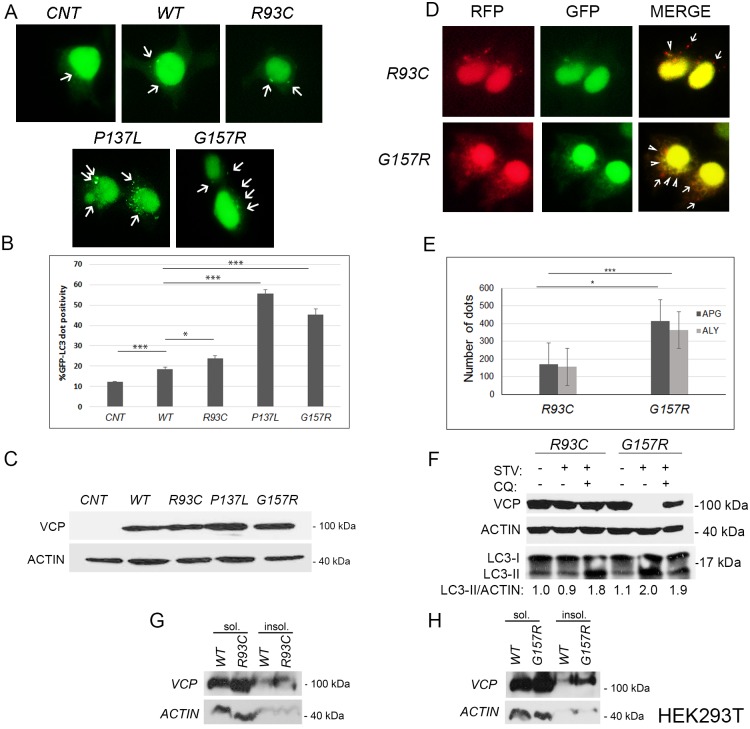
Another IBMPFD disease-associated mutant, the G157R mutant, was an autophagy target. (A-C) WT or mutant VCP/p97 constructs were co-transfected to HEK293T cells with GFP-tagged LC3 and autophagosomal dot formation was visualized (A) and quantified (B). Data were shown as mean ± SD of independent experiments (n = 3) ** p<0.01, *** p<0.001. (C) Example of an immunoblot showing the expression of WT or mutant VCP/p97 proteins in A and B. (D) HEK293T cells expressing VCP/p97 R93C or G157R mutants were co-transfected with RFP-GFP-LC3 fusion construct and dot formation was visualized under fluorescent microscope. Arrow heads indicate autophagosomes (yellow) and arrows indicate autolysosomes (red). (E) Quantification of autophagosome (APG) and autolysosome (ALY) numbers in cells expressing R93C or G157R mutants. Data were shown as mean ± SD of independent experiments (n = 3). *, p<0.05; ***, p<0.01 (F) Cells were transfected with MYC-tagged R93C or G157R mutant VCP/p97 constructs and starved (STV) or not in the absence or presence of the lysosomal inhibitor chloroquine (CQ, 10 μM, 40 min) and immunoblot analysis was performed. ACTIN was used as loading control. For LC3-II/ACTIN ratios band intensity ratios quantifications with the ImageJ software was used. (G and H) HEK293T expressing WT or mutant (R93C and G157R) VCP/p97 were fractionated, and subcellular distribution of soluble (sol.) and insoluble (insol.) proteins were analyzed by immunoblotting.

To determine whether the P137L mutant is the only VCP/p97 mutant that led to above-mentioned cellular outcomes, we also checked the effects of two other mutant proteins, namely VCP/p97 R93C and G157R mutants. Patients with R93C mutation as well as those with G157R mutation manifested all cardinal signs and symptoms of the disease, including muscle weakness, Paget’s disease of bone and cognitive decline [35]. In addition to these symptoms, two patients with the G157R mutation had early hearing impairment, a symptom that is uncommon to IBMPFD patients [5, 35]. Both R93C and G157R mutant proteins, were able to bind cofactors Npl4 and Ufd1 [17]. But only the G157R mutant was able to strongly induce autophagy as the P137L mutant did (Fig 7A–7C). Similar to observations with the P137L mutant, expression of the G157R mutant, but not the R93C mutant, led to a robust accumulation of both autophagosomes and autolysosomes (Fig 7D and 7E). Last but not least, starvation caused complete degradation of G157R mutant in a lysosomal activity-dependent manner (Fig 7F), while the R93C mutant protein levels were not affected under these conditions. Yet, G157R as well as R93C were found in both the soluble and the insoluble fraction of cells, the amount of insoluble G157R aggregates being more prominent (Fig 7G and 7H). Aggregation of the R93C mutant to a certain extent but lack of its degradation by autophagy might be the result of an autophagy defect caused by this mutant.

## Discussion

In this study, we showed that an IBMPFD-associated mutant VCP/p97 form, the P137L mutant protein is a target of macroautophagic degradation. We demonstrated that expression of the P137L mutant protein led to LC3 gel shift and GFP-LC3 dot formation under both basal and starvation conditions, highly indicative of autophagy activation. Moreover, in contrast with previous reports using other IBMPFD-related VCP/p97 mutants (R155 and R191), the P137L mutant did not interfere with autophagosome-lysosome fusion and autolysosomal function, shown here as follows: *i*.RFP-GFP-LC3 analyses showed a significant increase in both autophagosome and autolysosome numbers; *ii*.VCP/p97 P137L mutant protein formed cytosolic insoluble aggregates that colocalized with the autophagy marker GFP-LC3 and lysosomal protein LAMP1; *iii*. Addition of lysosomal inhibitors resulted in the accumulation of both LC3-II and mutant VCP/p97 under conditions activating autophagy (i.e. starvation and Torin-1 treatment). All these results showed that P137L mutant protein did not affect autophagosome maturation or lysosomal activity. Strikingly, P137L mutant protein formed aggregates that colocalized with the autophagy receptor p62 and that were targeted by autophagy and degraded by the autolysosomal activity. We also showed that another VCP/p97 mutant, G157R also formed aggregates that could be cleared by autophagy, underlining the fact that cellular phenotypes caused by P137L mutant expression were not isolated observations, and some other IBMPFD disease-related mutations might lead to similar outcomes.

IBMPFD is rare disease with an autosomal dominant inheritance and individuals from around 100 families were reported to suffer from the disease. Most common VCP/p97 mutations causing IBMPFD disease involved the R155 residue (around half of the reported cases). Consequently, almost all molecular studies were conducted using cells or animals expressing R155 mutant proteins as IBMPFD disease experimental models. We showed that, the P137L mutant VCP/p97 protein that was reported to cause IBMPFD diseasebehaved in a different way than the commonly studied mutant R155 [36][8]. In contrast with other VCP/p97 mutants, P137L mutant did not bind ERAD-related co-factors, and formed insoluble protein aggregates. Moreover, expression of the P137L mutant did not result in autophagosome maturation defects that were reported to be associated withthe VCP/p97 R155 mutant [21, 22]. In contrast, P137L protein aggregates colocalized with autophagosome and autolysosome markers, and they were degraded in autolysosomes, while WT and healthy VCP/p97 protein levels were maintained. We confirmed in our experimental system that R155C and R191Q mutants did not share the same faith with the P137L mutant. Strikingly, activation of autophagy was previously reported to aggravate the pathology in an R155C mutant mice model of the disease [37].

We showed that, the P137L mutant interacted with WT VCP/p97 proteins (S4 Fig). Since the mutant was unable to bind its cofactors and to contribute to VCP/p97-dependent cellular events such as ER-associated protein degradation (ERAD), hexamers containing the mutant protein should be non-functional. Indeed, upon expression of the P137L mutant, ubiquitylated protein accumulation was increased in tunicamycin-treated cells suffering from ER stress(S5 Fig). Moreover, we have previously shown that mutant proteins causing ER stress and ERAD, namely CFTRΔF508 and Tyrosinase C89R accumulated upon expression of the P137L mutant [17].

Binding of the p47 co-factor is important for VCP function in autophagic degradation. Yet, the P137L mutant was unable to bind this co-factor (probably due to a more detrimental alteration of VCP structure in this mutant) and the protein formed non-functional aggregates. Under these circumstances, mutant VCP/p97 proteins seemed not to interfere with the autophagy-related function of the endogenous wild-type VCP protein. Consequently, the wild-type VCP could still retain its function in autophagosome formation, and assist in the clearence of protein aggregates by autophagy, including P137L mutant aggregates. Therefore, in the case of IBMPFD-diseased individuals carrying the P137L mutation, WT VCP/p97 that is produced by the non-mutated allele might still allow autophagic degradation to proceed in an efficient manner.

**Fig 8:**
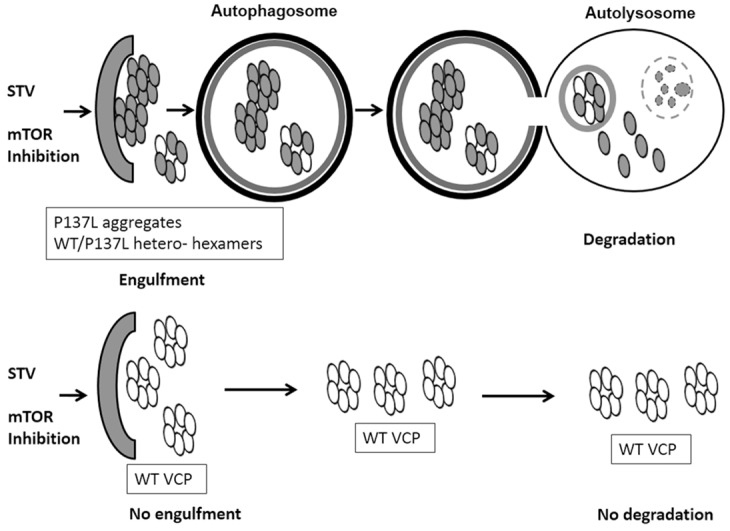
Model of P137 mutant vs WT VCP/p97 degradation. Following autophagy stimulation by starvation or Torin-1, aggregates containing VCP/p97 mutant P137L were sequestered in autophagosomes and delivered to autolysosomes for degradation. A soluble fraction of the mutant protein could interact with WT VCP/p97protein and form hetero-hexamers interfering with the function of the WT protein. On the other hand, a major fraction of the WT VCP/p97was not colocalizing with autophagosomes and autolysosomes, and it was not significantly degraded by the autolysosomal activity. Therefore, autophagy activation led to a selective degradation of the mutant protein, while the WT counterpart was spared.

In IBMPFD disease,P137L mutant formed protein aggregates that did not prominently interfere with autophagic degradation activity. But, ERAD function was still perturbed and ubiquitylated proteins accumulated (S5 Fig), resulting in the perturbation of normal cellular functions [38]. In this context, preferential autophagic clearance of the mutant proteinallowedthe enrichment of WT and functional VCP/p97 complexes (Fig 8). Therefore, autophagy activation in a subset of patients suffering from the IBMPFD disease might be beneficial and autophagy-based treatment of these patients might be possible. Further studies will reveal whether observations made here in *in vitro* cell culture conditions are valid in *in vivo* animal models.

Recently, VCP/p97 was shown to contribute to the autophagy of mitochondria (mitophagy) [39, 40]. Yet in our system, expression of the P137L mutant did not prominently affect mitophagy compared to WT VCP/p97 under basal conditions, and even after treatment of cells with the mitophagy inducer CCCP (S6 Fig). These results show that neither autophagy nor mitophagy was defective in cells expressing the mutant protein.

VCP/p97 positive aggregates are not specific for IBMPFD. In a wide variety of diseases such as Parkinson’s disease, Hungtinton’s disease, Lewy Body disease, ALS and Machado-Joseph disease, VCP/p97 positive aggregates were observed [41–43]. Moreover, mutations of VCP/p97 were reported in a fraction of ALS patients [44]. Whether the P137L mutation and similar mutations or conditions causing aggregation of the VCP/p97 protein contribute to the pathologies observed in above-mentioned diseases is of interest. Large-scale genetic analysis of patients suffering from IBMPFD and other more common diseases will reveal the frequency of such mutations and the impact of the molecular events described in this study on disease progression.

Although all VCP/p97 mutations gave rise to clinical signs and symptoms of the IBMPFD disease, we showed that molecular and cellular changes caused by different VCP/p97 mutants (R155, R191, R93 mutants versus P137 and G157 mutants) were not identical. Our study underlines the fact that caution should be exerted when modeling a disease based on single or few common mutations. Therefore, before designing a single common therapy approach for all patients regardless of individual genetic changes, a better understanding of the biology of various disease-associated mutant proteins should be obtained.

## Supporting Information

S1 Fig VCP/p97 P137L mutant induced both autophagosome and autolysosome formation in U2OS and PC-12 cells. (A and C) Cells expressing empty vector wild type (WT), mutant (P137L or R155C) VCP/p97 were co-transfected with RFP-GFP-LC3 fusion construct, treated with chloroquine (CQ) for 40 min and dot formation was visualized in U2OS (A) and PC-12 (C) cells. Arrow heads indicate autophagosomes whereas arrows indicate autolysosomes. (A and C) The graphs show the number of autophagosomes and autolysosomes in U2OS (B) and PC-12 (D) cells under basal and chloroquine (CQ) treated condition. Data were shown as mean ± SD of independent experiments (n = 3). * p<0.05, ** p<0.01, *** p<0.001.
(PDF)

S2 Fig Mutant VCP/p97 formed aggregates and it was mainly found in the insoluble fraction of cells. (A) HEK293T cells were transfected with wild type (WT) and mutant (P137L or R155C) VCP/p97 and then analyzed under confocal microscopy. (B) Mouse primary myoblast cells were cotransfected with autophagosome marker GFP-LC3 and wild type (WT) or mutant (P137L) VCP/p97 and analyzed using confocal microscopy under basal (NON-STV) or starved (STV) conditions. (C-E) Cells expressing WT or mutant (P137L or R155C) VCP/p97 were fractionated and subcellular distribution of proteins were analyzed by immunoblotting in HEK293T (C), U2OS (D) or PC-12 (E) cells. Graphs show the percentage of the insoluble fraction over total protein amount. ACTIN was used as loading control. (F) Wild type (WT) and mutant P137L VCP/p97 expressing U2OS cells transfected with a GFP-p62 construct, and association between p62 (Green) and VCP (Red) was analyzed under confocal microscopy.
(PDF)

S3 Fig P137L mutant VCP/p97 is not a short-lived protein. U2OS cells were transfected with wild type (WT) or mutant (P137L) VCP/p97 in the absence or presence of 25 μM cycloheximide (CHX) for indicated time points. Western blot analysis was performed by using MYC and ACTIN antibodies. ACTIN was used as loading control. Image J software was used for the quantification of band intensities.
(PDF)

S4 Fig P137L mutant can bind to WT VCP/p97. (A)MYC-tagged wild type (WT) or mutant (P137L) were transfected to HEK293T cells. VCP proteins were precipitated using MYC-beads and immunoblots were incubated with anti-MYC or anti-VCP/p97 antibodies. ACTIN was used as loading control. (B) U2OS and (C) PC-12 cells expressing MYC-tagged wild type (WT) or mutant (P137L) were co-transfected with GFP-tagged wild type VCP/p97. Myc-tagged WT and P13L VCP proteins were immunoprecipitated using MYC-beads and blots were incubated with GFP, VCP/p97 and ACTIN antibodies. ACTIN was used as oading control.
(PDF)

S5 Fig P137L mutant expression led to ubiquitylated protein accumulation. (A) WT and (B) P137L mutant VCP expressing cells were treated with 1 μg/ml Tunicamycin for indicated time points (0 to 8 hours). An anti-ubiquitin antibody was used to detect ubiquitylated total proteins. ACTIN was used as loading control.
(PDF)

S6 Fig VCP/p97P137L mutant expression did not interfere with mitophagy. (A) HEK293T cells expressing were co-transfected wild type (WT) or mutant (P137L) VCP/p97 and autophagy marker GFP-LC3 and mitochondrial marker Mito-dsRed. 10 μM CCCP treatment for 12 h was used as mitophagy inducer and DMSO as carrier. Mitophagy was assessed as GFP-LC3-Mito-dsRed colocalization under confocal microscope. (B) Quantification of mitophagy positive cells (mean ± S.D. of independent experiments, n = 3. For each condition, 30 cells per point were counted. ** p<0.01, *** p<0.001, ns, not significant).
(PDF)

## Abbreviations

VCP/p97: valosin containing protein
WT: wild type
P137L: Pro-137 to Leu
R93C: Arg-93 to Cys
R155C: Arg-155 to Cys
G157R: Gly-157 to Arg
R155C: Arg-155 to Cys
R191Q: Arg-191 to Gln
LC3: Microtubule-associated protein 1A/1B-light chain 3
p62: sequestosome 1
IBMPFD: Inclusion body myopathy with early-onset Paget disease and frontotemporal dementia
CQ: chloroquine
stv: starvation
TRN: Torin-1
