# Correction: IBMPFD Disease-Causing Mutant VCP/p97 Proteins Are Targets of Autophagic-Lysosomal Degradation

**DOI:** 10.1371/journal.pone.0167192

**Published:** 2016-11-18

**Authors:** The PLOS ONE Staff

The authors Ozlem Oral, Nur Mehpare Kocaturk, and Devrim Gozuacik should not have been attributed equal contribution to this work. Only Ozlem Oral and Nur Mehpare Kocaturk should be listed as co-second authors and contributing equally to this work. Devrim Gozuacik should only be listed as the Corresponding Author for this work. The publisher apologizes for this error.
