# Supplementary figures and images for: IBMPFD Disease-Causing Mutant VCP/p97 Proteins Are Targets of Autophagic-Lysosomal Degradation
Source: PLoS One. 2016 Oct 21;11(10):e0164864. doi: 10.1371/journal.pone.0164864 (PMC5074563; doi:10.1371/journal.pone.0164864)

S1

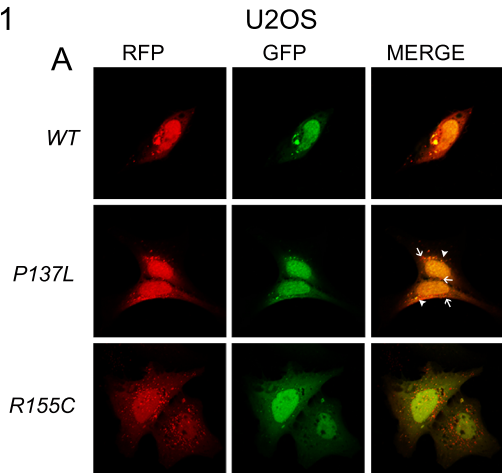

B

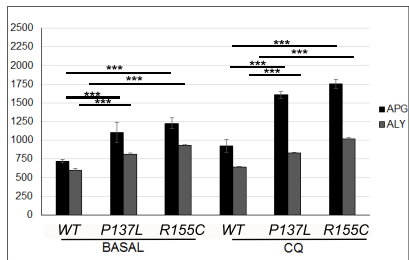

PC-12

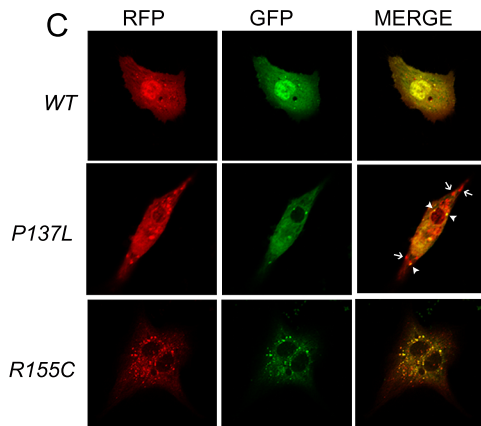

D

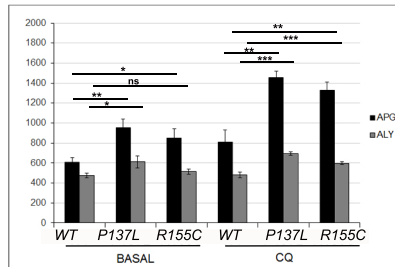

Supplement: S1 Fig — (A and C) Cells expressing empty vector wild type (WT), mutant (P137L or R155C) VCP/p97 were co-transfected with RFP-GFP-LC3 fusion construct, treated with chloroquine (CQ) for 40 min and dot formation was visualized in U2OS (A) and PC-12 (C) cells. Arrow heads indicate autophagosomes whereas arrows indicate autolysosomes. (A and C) The graphs show the number of autophagosomes and autolysosomes in U2OS (B) and PC-12 (D) cells under basal and chloroquine (CQ) treated condition. Data were shown as mean ± SD of independent experiments (n = 3). * p<0.05, ** p<0.01, *** p<0.001. (PDF) [file pone.0164864.s001.pdf]

# S3

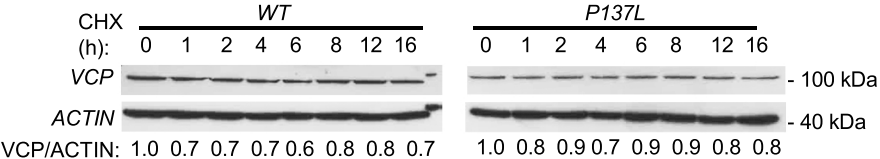

Supplement: S3 Fig — U2OS cells were transfected with wild type (WT) or mutant (P137L) VCP/p97 in the absence or presence of 25 μM cycloheximide (CHX) for indicated time points. Western blot analysis was performed by using MYC and ACTIN antibodies. ACTIN was used as loading control. Image J software was used for the quantification of band intensities. (PDF) [file pone.0164864.s003.pdf]

# S4

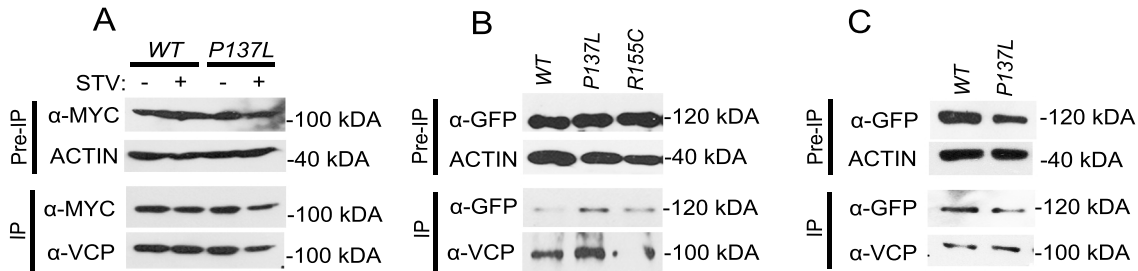

Supplement: S4 Fig — (A)MYC-tagged wild type (WT) or mutant (P137L) were transfected to HEK293T cells. VCP proteins were precipitated using MYC-beads and immunoblots were incubated with anti-MYC or anti-VCP/p97 antibodies. ACTIN was used as loading control. (B) U2OS and (C) PC-12 cells expressing MYC-tagged wild type (WT) or mutant (P137L) were co-transfected with GFP-tagged wild type VCP/p97. Myc-tagged WT and P13L VCP proteins were immunoprecipitated using MYC-beads and blots were incubated with GFP, VCP/p97 and ACTIN antibodies. ACTIN was used as oading control. (PDF) [file pone.0164864.s004.pdf]

S5

A

*WT*TUN  
(h):

0 1 2 4 8 16

UBQ

-130 kDa  
-110 kDa  
-70 kDa  
-55 kDa

ACTIN

-40 kDa

B

P137L

TUN  
(h):

0 1 2 4 8 16

UBQ

-130 kDa  
-110 kDa  
-70 kDa  
-55 kDa

ACTIN

-40 kDa

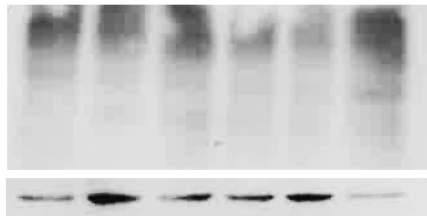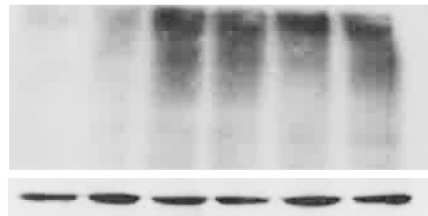

Supplement: S5 Fig — (A) WT and (B) P137L mutant VCP expressing cells were treated with 1 μg/ml Tunicamycin for indicated time points (0 to 8 hours). An anti-ubiquitin antibody was used to detect ubiquitylated total proteins. ACTIN was used as loading control. (PDF) [file pone.0164864.s005.pdf]

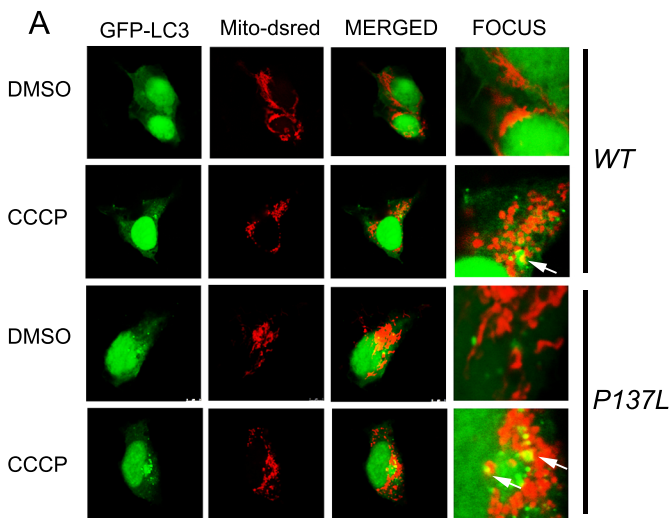

**B**

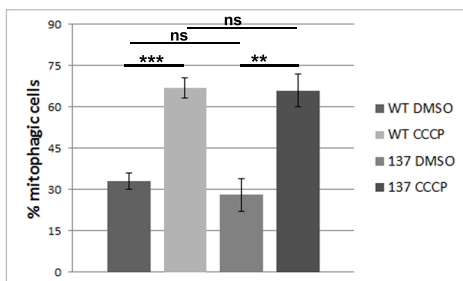

Supplement: S6 Fig — (A) HEK293T cells expressing were co-transfected wild type (WT) or mutant (P137L) VCP/p97 and autophagy marker GFP-LC3 and mitochondrial marker Mito-dsRed. 10 μM CCCP treatment for 12 h was used as mitophagy inducer and DMSO as carrier. Mitophagy was assessed as GFP-LC3-Mito-dsRed colocalization under confocal microscope. (B) Quantification of mitophagy positive cells (mean ± S.D. of independent experiments, n = 3. For each condition, 30 cells per point were counted. ** p<0.01, *** p<0.001, ns, not significant). (PDF) [file pone.0164864.s006.pdf]
