# Supplementary material for: IBMPFD Disease-Causing Mutant VCP/p97 Proteins Are Targets of Autophagic-Lysosomal Degradation
Source: PLoS One. 2016 Oct 21;11(10):e0164864. doi: 10.1371/journal.pone.0164864 (PMC5074563; doi:10.1371/journal.pone.0164864)
Supplement: S2 Fig — (A) HEK293T cells were transfected with wild type (WT) and mutant (P137L or R155C) VCP/p97 and then analyzed under confocal microscopy. (B) Mouse primary myoblast cells were cotransfected with autophagosome marker GFP-LC3 and wild type (WT) or mutant (P137L) VCP/p97 and analyzed using confocal microscopy under basal (NON-STV) or starved (STV) conditions. (C-E) Cells expressing WT or mutant (P137L or R155C) VCP/p97 were fractionated and subcellular distribution of proteins were analyzed by immunoblotting in HEK293T (C), U2OS (D) or PC-12 (E) cells. Graphs show the percentage of the insoluble fraction over total protein amount. ACTIN was used as loading control. (F) Wild type (WT) and mutant P137L VCP/p97 expressing U2OS cells transfected with a GFP-p62 construct, and association between p62 (Green) and VCP (Red) was analyzed under confocal microscopy. (PDF) [file pone.0164864.s002.pdf]

S2

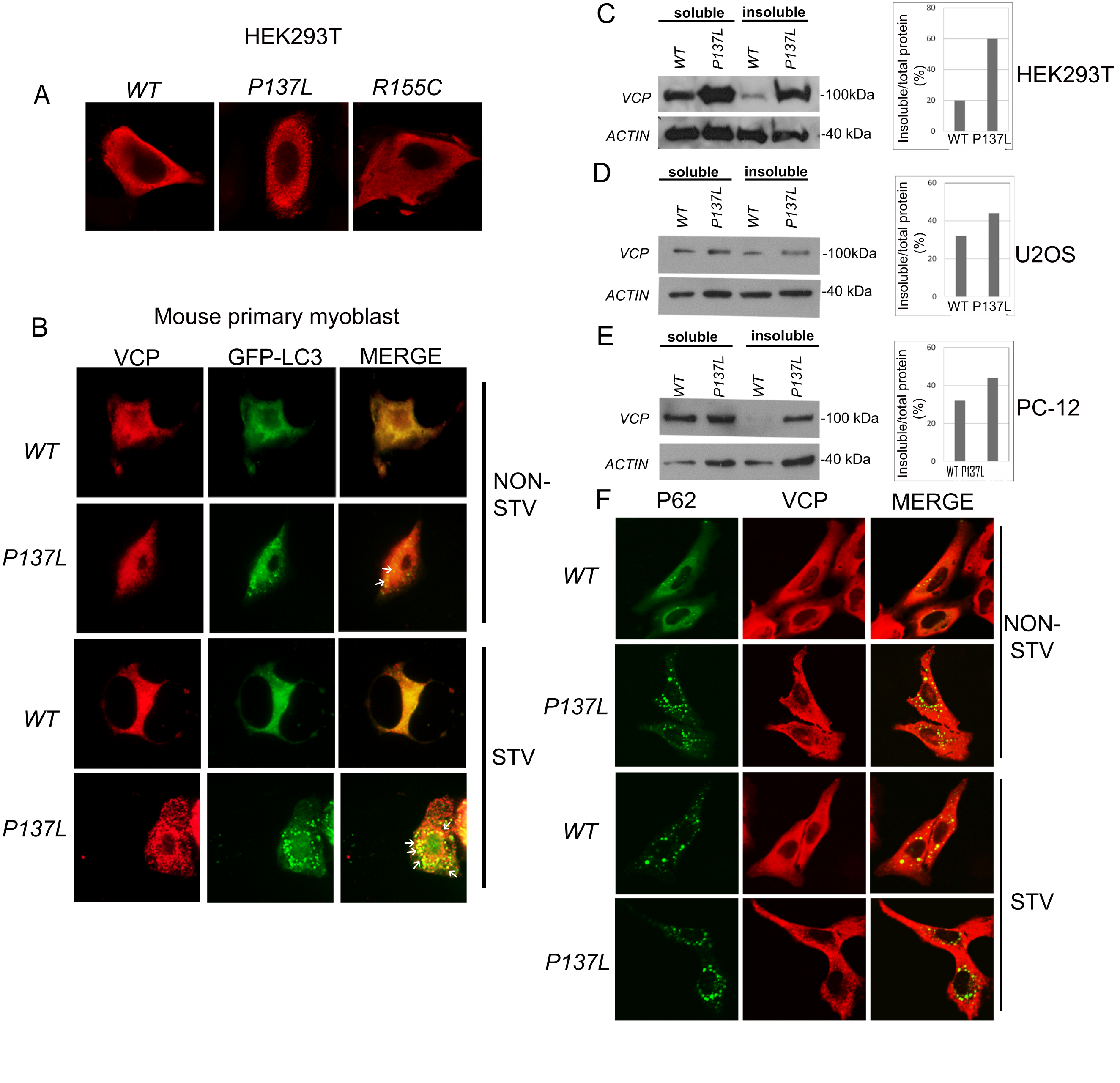

HEK293T
A
WT
P137L
R155C
B
Mouse primary myoblast
VCP
GFP-LC3
MERGE
WT
P137L
WT
P137L
NON-STV
STV
C
soluble
insoluble
WT
P137L
WT
P137L
VCP
ACTIN
-100kDa
-40 kDa
Insoluble/total protein (%)
WT P137L
HEK293T
D
soluble
insoluble
WT
P137L
WT
P137L
VCP
ACTIN
-100kDa
-40 kDa
Insoluble/total protein (%)
WT P137L
U2OS
E
soluble
insoluble
WT
P137L
WT
P137L
VCP
ACTIN
-100 kDa
-40 kDa
Insoluble/total protein (%)
WT P137L
PC-12
F
P62
VCP
MERGE
WT
P137L
WT
P137L
NON-STV
STV
